# Equality in healthcare: transcultural psychiatry

**DOI:** 10.1192/j.eurpsy.2022.1626

**Published:** 2022-09-01

**Authors:** C. Alvarez Garcia, A. Gomez Martín

**Affiliations:** Hospital Universitario Príncipe de Asturias, Psychiatry, Alcalá de Henares, Spain

**Keywords:** migrants, transcultural, mental healthcare

## Abstract

**Introduction:**

Migratory flows are increasing more and more, especially regarding the refugee crisis during the last years. There are around 86,7 million migrants in Europe. Migrants share similar experiences that may affect their physical and mental health, such as loss of a social network, lack of economical support or high levels of stress and discrimination.

**Objectives:**

To analyze the obstacles that migrants must face to obtain a mental health assistance and the importance of an intercultural approach.

**Methods:**

A narrative review of the existing literature on the subject.

**Results:**

Although there exists evidence that shows that migrants tend to have more health needs, they usually seek less medical advice and receive a poor-quality attention, fulfilling the inverse-care law. This is due to several reasons. Many migrants are excluded of the health care system due to bureaucratic impediments. Also, the language has a determining role, since a higher quality of communication could lead to a better understanding of the symptoms, reducing the risk of erroneous evaluations. Besides, different background and culture between the patient and the doctor can result in lack of communication, mistrust, mistreatment, poor adherence, and worse prognosis.

**Conclusions:**

Despite the exponential growth of migration in the last decade and the continue progression, migrants still face many barriers to receive healthcare. It is necessary to do more research on the mental health of migrants and ethnic minorities to ensure quality care to different cultures.

**Disclosure:**

No significant relationships.

